# Biological and Evolutionary Significance of Terminal Extensions of Mitochondrial Translation Initiation Factor 3

**DOI:** 10.3390/ijms19123861

**Published:** 2018-12-04

**Authors:** Ksenia Derbikova, Anton Kuzmenko, Sergey Levitskii, Maria Klimontova, Ivan Chicherin, Maria V. Baleva, Igor A. Krasheninnikov, Piotr Kamenski

**Affiliations:** 1 Faculty of Biology, M.V. Lomonosov Moscow State University, 119991 Moskva, Russia; k.derbikova@gmail.com (K.D.); ms-ms@list.ru (A.K.); krolick@yandex.ru (S.L.); m.klimontova@gmail.com (M.K.); i.v.chicherin@gmail.com (I.C.); mary-bw@mail.ru (M.V.B.); iakrasheninnikov@protein.bio.msu.ru (I.A.K.); 2 Institute of Molecular Genetics, Russian Academy of Science, 119991 Moskva, Russia; 3 Faculty of Biosciences, Heidelberg University, 69117 Heidelberg, Germany

**Keywords:** mitochondria, translation, initiation, initiation factor, terminal extension

## Abstract

Protein biosynthesis in mitochondria is organized in a bacterial manner. However, during evolution, mitochondrial translation mechanisms underwent many organelle-specific changes. In particular, almost all mitochondrial translation factors, being orthologous to bacterial proteins, are characterized by some unique elements of primary or secondary structure. In the case of the organellar initiation factor 3 (IF3), these elements are several dozen amino acids long N- and C-terminal extensions. This study focused on the terminal extensions of baker’s yeast mitochondrial IF3, Aim23p. By in vivo deletion and complementation analysis, we show that at least one extension is necessary for Aim23p function. At the same time, human mitochondrial IF3 is fully functional in yeast mitochondria even without both terminal extensions. While *Escherichia coli* IF3 itself is poorly active in yeast mitochondria, adding Aim23p terminal extensions makes the resulting chimeric protein as functional as the cognate factor. Our results show that the terminal extensions of IF3 have evolved as the “adaptors” that accommodate the translation factor of bacterial origin to the evolutionary changed protein biosynthesis system in mitochondria.

## 1. Introduction

Mitochondria are essential organelles for virtually all eukaryotic cells. They possess many important functions such as ATP production, apoptosis management, amino acids and fatty acids biosynthesis. Mitochondria are thought to originate from an ancient bacteria about 1–2 billion years ago. During evolution, mitochondrial genomes have been strongly reduced with the majority of genes migrated to the nucleus. The protein products of these genes are imported to the organelles from the cytosol [1]. However, the modern mitochondria still contain genomes of a small size (from ~10^4^ to ~10^6^ nucleotides, depending on the species) that code for several proteins and RNAs. Correspondingly, all mechanisms of gene expression are realized in mitochondria.

Mitochondrial translation is generally organized in a prokaryotic manner. However, this process is characterized by several specific features and its molecular mechanisms are species-specific to a great extent [2,3]. Recent cryo-electron microscopy studies of the yeast and mammalian mitochondrial ribosomes have revealed their high structural divergence from the bacterial ribosomes [4,5]. Mechanistically, the majority of the mitochondria-specific properties of translation seem to be concentrated on the initiation stage [6,7] and these properties are different among species. In yeast, it is worth mentioning that mitochondrial translation initiation is possible even if the initiator tRNA^Met^ is not formylated [8]. One more curious feature of this process in yeast mitochondria is the presence of a unique system of so-called translational activators. These are proteins that specifically bind a given mitochondrial mRNA, thus, making its translation possible; they also link together the processes of translation and respiratory complexes assembly [9]. With regard to mammalian mitochondrial translation, a single tRNA^Met^ species functions there as the initiator as well as the elongator tRNA and only those molecules that are formylated can take part in the initiation [10]. Besides that, mammalian mitochondrial mRNAs do not possess any 5′-untranslated regions (UTRs) [11].

Regardless of species, the system of mitochondrial translation initiation factors is different from that in bacteria, where three initiation factors exist, namely IF1, IF2 and IF3. Each of them is obligatory for correct translation. In mitochondria, however, only one of the orthologous factors, mitochondrial IF2 (mtIF2), has robustly been identified among species. IF1 as an individual polypeptide is absent in the mitochondria; its function is performed by a short insertion domain of mtIF2 [12]. In mammals, this domain has been shown to close the decoding center during initiation and to enhance binding of a leaderless mRNA to ribosome [7]. The insertion domain of mtIF2 is conserved only in vertebrate animals; the question about IF1 function in mitochondria of invertebrate organisms is still unresolved [13]. Concerning mitochondrial IF3 (mtIF3), this factor has been identified and characterized in mammals and several other species [14]. On the other hand, in some fungi and other lower eukaryotes, mtIF3 had not been found for a while. In particular, mtIF3 has not been identified in baker’s yeast *Saccharomyces cerevisiae* until 2012 when a good candidate was found using bioinformatical approaches [13]. This candidate was Aim23p, a protein with mitochondrial localization and unknown function. With the help of complementation experiments in yeast cells (exchanging the *AIM23* gene by annotated gene of mtIF3 from human cells or fission yeast *Schizosaccharomyces pombe*), it has been genetically proven that Aim23p indeed functions as mtIF3 [6,13]. However, it has been revealed later that Aim23p takes part in mitochondrial translation in some non-canonical ways; deletion of the *AIM23* gene does not result in protein biosynthesis arrest in mitochondria, which is quite unexpected taking into account that in all known translation systems initiation factor 3 is indispensable. Instead, mitochondrial translation in yeast becomes “misbalanced” in the absence of Aim23p; the amount of several proteins becomes lower while the synthesis of some other proteins goes even faster [15].

As do its bacterial orthologues, mtIF3 contains N- and C-terminal domains connected by an unstructured linker. Mammalian mtIF3 was shown to catalyze the formation of the initiation complex in vitro in the presence of mitochondrial ribosomes, mtIF2, mitochondrial initiator tRNA, and mRNA [14]. In addition, mammalian mtIF3 has an affinity to the small mitoribosomal subunit and promotes the dissociation of mitoribosomes (55S) towards separated small (28S) and large (39S) subunits [14] (known as ribosome splitting). The mode of splitting by mtIF3 is active, which means that the protein does not simply shift the equilibrium between 55S mitoribosomes and separated 39S and 28S subunits by binding to a free 28S and preventing further subunits re-association. Instead, mammalian mtIF3 binds to 55S mitoribosomes and promotes their dissociation, probably through the formation of a transient intermediate, which rapidly partitions to the 28S subunit complexed with mtIF3 and free 39S subunit [16]. Besides, mammalian mtIF3 has one more function, which does not pertain to its bacterial orthologue, namely promoting the dissociation of initiator tRNA from mitoribosomes in the absence of mRNA [17].

Mitochondria-specific regions of mammalian mtIF3 are short (about 30 amino acids each) N- and C-terminal extensions located in the very distal parts of the protein. These extensions also have been extensively studied by analyzing different mutant versions of mammalian mtIF3 in several in vitro assays. Deletion of these extensions makes the protein slightly more active in the promotion of the initiation complex formation [17]. The mammalian mtIF3 without N- and C-terminal extensions binds to the 28S subunit with the same affinity as the wild-type protein. However, the affinity of the truncated protein to the 39S subunit is one order higher than that of the wild-type mtIF3 [18]. Such high affinity leads to incorrect joining of the 39S subunit to the initiation complex and to a mtIF3 partial arrest in complex with 55S mitoribosomes after subunits association. These results have led to a hypothesis according to which the mammalian mtIF3 terminal extensions have mainly evolved in order to reduce the factor affinity to the large mitoribosome subunit [18]. Finally, the above-mentioned mitochondria-specific activity of mammalian mtIF3 (evacuating the initiator tRNA from mitoribosomes if mRNA is not bound) is assured by its C-terminal extension [17].

According to an in silico analysis, the topology of baker’s yeast mtIF3, Aim23p, is quite close to that of mammalian mtIF3 [13] with all of the protein domains conserved. In particular, the N- and C-terminal extensions are also present in Aim23p, although being slightly longer than in the case of mammalian mtIF3. This work is dedicated to the functional analysis of Aim23p terminal extensions. Using methods of yeast genetics, we have shown in vivo that either the N- or C-terminal extension of Aim23p is enough for normal mitochondrial translation. Moreover, *E. coli* IF3 versions fused either with the N-terminal extension of Aim23p or with both extensions are as active in yeast mitochondria as the wild-type Aim23p. Finally, we have demonstrated that human mtIF3 is functionally active in yeast mitochondria even without the terminal extensions. These experimental facts have allowed us to hypothesize that the terminal extensions of mitochondrial IF3 might possess different functions in different species.

## 2. Results

### 2.1. At Least One Terminal Extension of Aim23p is Necessary for Its Function in Mitochondria

**Figure 1:**
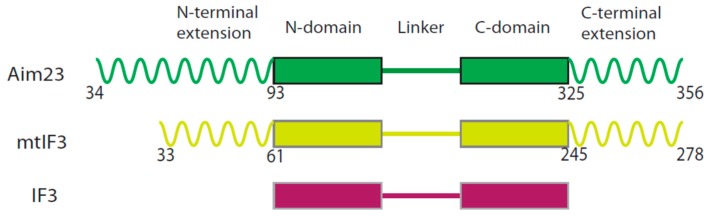
Domain organization of translation initiation factors 3 from yeast mitochondria (Aim23p, green), human mitochondria (mtIF3, yellowish green), and *E. coli* (IF3, dark red). Mitochondrial targeting sequences (MTSs) of Aim23p and mtIF3 are not shown as they are absent in the mature, functionally active protein forms. The main structural regions of the proteins are indicated on the top. Helices correspond to the terminal extensions, boxes correspond to the N- and C-domains and lines correspond to the linker regions. Characters are numbers of the amino acids circumscriptive of the indicated structural regions.

As mentioned above, the overall topology of Aim23p resembles that of mammalian mtIF3. According to the in silico structure prediction [13], Aim23p consists of an N-terminal mitochondrial targeting sequence followed by a 60 amino acid long N-terminal extension, N-domain, linker, C-domain, and a 32 amino acid long C-terminal extension (Figure 1).

**Figure 2:**
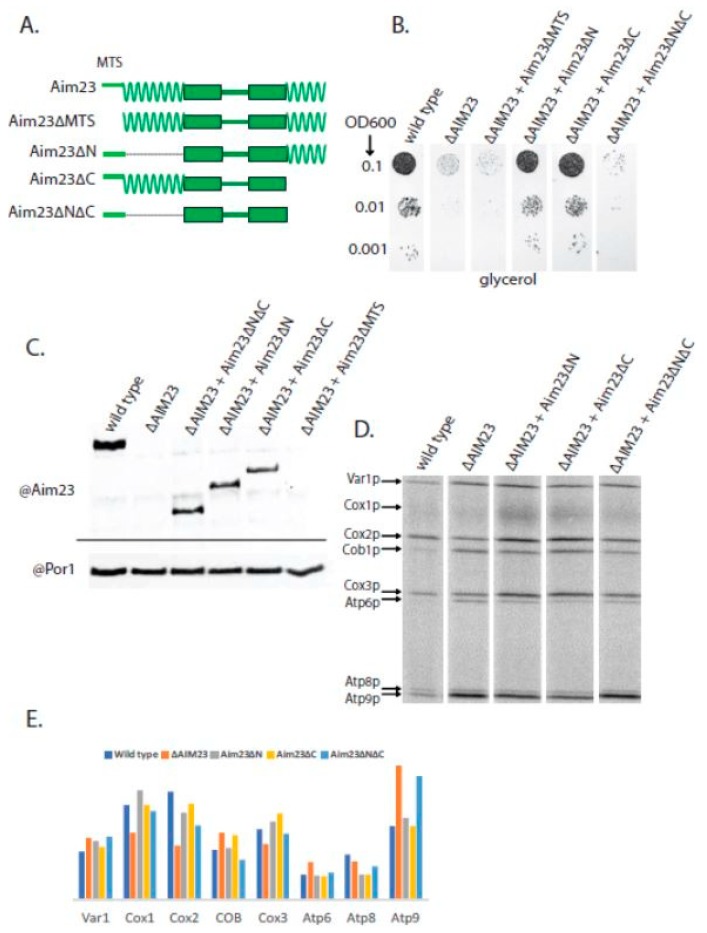
At least one terminal extension is required for proper Aim23p function in yeast mitochondria. (**A**) Scheme of the hybrid proteins (the names are indicated on the left). The designations of the main structural parts and their colors are the same as in Figure 1. (**B**) The plate with a glycerol-containing medium after 2 days of growth of the yeast strains indicated on the top. The ten-fold dilutions of the yeast suspensions were plated, starting from OD600 = 0.1 (indicated on the left). Wild-type: D273-10B with the *AIM23* gene genomic disruption and with the Aim23p-coding plasmid. ∆AIM23: D273-10B with the *AIM23* genomic disruption containing the empty vector. All other strains: D273-10B with the *AIM23* genomic disruption supplemented with the corresponding mutant Aim23p version encoded by the plasmid. The experiment has been biologically repeated three times; the characteristic result is presented. (**C**) Western-blot hybridization of mitochondrial proteins from the above-mentioned strains with anti-Aim23p antibodies (top part). As a loading control, the same samples were blotted with anti-porin 1 antibodies (bottom part). (**D**) Separation of radioactively-labeled products of mitochondrial translation of the yeast strains indicated on the top. Cytosolic translation was inhibited by cycloheximide, which was followed by the addition of ^35^S-methionine incorporated exclusively in the mitochondrially-encoded proteins (for details, see Materials and Methods). The bands corresponding to all of the eight individual proteins encoded in yeast mitochondria are visible; the proteins’ names are indicated on the left. (**E**) Estimated levels of the mitochondrially-encoded proteins (indicated on the bottom) after 15 min of labeling with ^35^S-methionine in the yeast strains indicated on the top.

In order to assess the importance of the terminal extensions for Aim23p function, we constructed three mutant versions of this protein without the N-terminal extension (Aim23∆N), without the C-terminal extension (Aim23∆C), and without both extensions (Aim23∆N∆C). In all three proteins, the predicted mitochondrial targeting sequence (MTS) has been kept since it is obligatory for protein to be imported into the mitochondria. This targeting sequence should be cut after the import into the mitochondrial matrix [19] producing the mature, active protein. However, the exact length of the Aim23p MTS has never been verified experimentally. To ensure that the computer prediction of the MTS was correct, we constructed a fourth mutant version of Aim23p, with both terminal extensions but without the predicted MTS (Aim23∆MTS). A schematic representation of the mutant Aim23p versions may be found in Figure 2A.

Earlier, we constructed a yeast strain where the *AIM23* gene was disrupted (further referred to as ∆AIM23) [15]. Surprisingly, this disruption did not lead to the complete arrest of mitochondrial translation; rather protein biosynthesis was misbalanced. The mitochondrial function of this strain was altered only during the first 1–2 days of incubation, after which normal organellar work restored. The easiest way of assessing the mitochondrial function in yeast is to let them grow on a medium with a non-fermentable carbon source (such as glycerol, ethanol, or lactate) and to measure the rate of this growth. In the case of the ∆AIM23 strain, there was almost no growth on a glycerol-containing agarized medium after the first two days followed by complete restoration of the growth rate up to the wild-type level over the next 1–2 days [15]. In this work, we transformed the ∆AIM23 strain with four different plasmids coding for the above-described mutant versions of Aim23p. After clone selection, we tested the ability of the resulting strains to grow on a glycerol-containing medium for 2 days (Figure 2B). Such growth time was chosen in order to have an opportunity to clearly distinguish between the wild-type and ∆AIM23 phenotypes. As a control, the same strains were dropped on the glucose-containing medium which, as expected, gave no differences between the wild-type and mutant strains since yeast growth on glucose does not depend on mitochondrial function. The presence of the mutant proteins in yeast mitochondria was also controlled. For that, we did Western-blot hybridization of mitochondrial extracts with antibodies against Aim23p (Figure 2C). All Aim23p versions were indeed detected in the mitochondria, except for the Aim23∆MTS. This last observation was expected since Aim23∆MTS cannot be imported into the organelle due to the absence of the mitochondrial targeting sequence.

Figure 2B shows that the ∆AIM23 strain almost does not grow after 2 days of incubation on a glycerol medium, which is in perfect accordance with the results obtained earlier. At the same time, the presence of the Aim23p versions without either an N- or C-terminal extension in the ∆AIM23 strain results in a growth rate equal to that of the wild-type yeast. Aim23p without both extensions, however, does not increase the growth rate of the ∆AIM23 strain to any visible extent. Finally, if MTS is absent in Aim23p, such protein also does not help the growth of ∆AIM23 on glycerol. Thus, at least one terminal extension in Aim23p is sufficient for the protein to be fully functional in yeast mitochondria. The absence of both extensions together makes Aim23p incapable of maintaining mitochondrial function. This, in turn, indicates the functional importance of the N- and C-terminal extensions of Aim23p, in full agreement with the results obtained for the mammalian mtIF3 in in vitro experiments (see Introduction).

In order to assess the influence of the above-described Aim23p mutations on the mitochondrial translation in more detail, we analyzed the profiles of protein biosynthesis products in the mitochondria of mutant yeast strains. For that, we added cycloheximide to the cultured cells, which completely blocked cytosolic translation but did not affect this process in mitochondria. Thereafter, we supplied yeast cultures with radiolabeled ^35^S-methionine, which could incorporate only in the mitochondrially-synthesized polypeptide chains [20]. The result of the radiolabeled mitochondrial proteins separation is shown in Figure 2D. In our previous work, we performed the analogous experiment with wild-type and ∆AIM23 strains and found that the absence of Aim23p had no meaningful effect on the amount of Var1p and led to a decrease in the amount of Cox1p and Cox3p and, surprisingly, to a significant increase in the amount of Atp6p and Atp9p [15]. This is exactly what we have obtained in this work (Figure 2E). Speaking about mutant strains containing truncated Aim23p versions, we have not revealed a solid correlation between their growth on glycerol and the rate of individual mitochondrial proteins synthesis. The levels of Cox1p, Cox2p and Atp6p were very similar in these strains, all being closer to those of the wild-type. However, the Atp9p synthesis rate was increased in Aim23∆N∆C (like in ∆AIM23 strain) and was not affected in the Aim23∆N and Aim23∆C strains, which perfectly fits the results of the glycerol growth experiment. Thus, the presence of at least one extension ensures the functionality of the mitochondria.

### 2.2. Terminal Extensions of Aim23p Make E. coli IF3 Fully Active in Yeast Mitochondria

**Figure 3:**
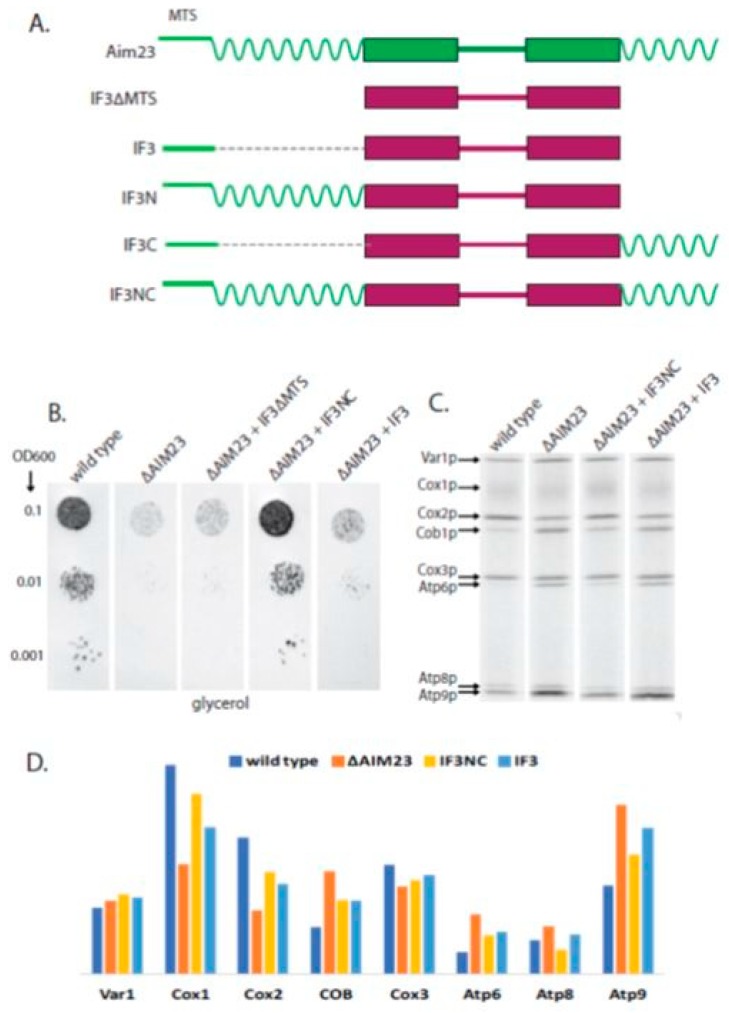
Aim23p terminal extensions fused to bacterial IF3 restore the biological activity of the chimeric protein in yeast cells. (**A**) Scheme of the chimeric proteins (the names are indicated on the left). The designations of the main structural parts and their colors are the same as in Figure 1. (**B**) The plate with a glycerol-containing medium after 2 days of growth of the yeast strains indicated on the top. The ten-fold dilutions of the yeast suspensions were plated, starting from OD600 0.1 (indicated on the left). Wild-type and ∆AIM23: see Figure 2 legend. All other strains: D273-10B with the *AIM23* genomic disruption supplemented with the corresponding mutant Aim23p version encoded by the plasmid. The experiment has been biologically repeated three times; the characteristic result is presented. (**C**) Separation of radioactively-labeled products of mitochondrial translation of the yeast strains indicated on the top. Cytosolic translation was inhibited by cycloheximide which was followed by adding of ^35^S-methionine incorporated exclusively in the mitochondrially-encoded proteins (for details, see Materials and Methods). The bands corresponding to all the eight individual proteins encoded in yeast mitochondria are visible; the proteins’ names are indicated on the left. (**D**) Estimated levels of the mitochondrially-encoded proteins (indicated on the bottom) after 15 min of labeling with ^35^S-methionine in the yeast strains indicated on the top.

We have previously shown that *E. coli* IF3 with the MTS of Aim23p, being synthesized in yeast cells instead of Aim23p, provides little but meaningful functional activity to the mitochondria: faster growth of the corresponding strain on glycerol medium has been observed compared to the ∆AIM23 strain [6]. This is a powerful genetic evidence of the fact that Aim23p is indeed mtIF3. We also have demonstrated that human mtIF may successfully substitute for Aim23p in yeast mitochondria [6]. To assess the biological functions of Aim23p terminal extensions, we addressed the question if they can increase the activity of *E. coli* IF3 with MTS of Aim23p in yeast mitochondria. To answer this question, we constructed another set of mutant proteins based on IF3 (Figure 3A). The initial idea was to fuse this protein with either N-terminal extension of Aim23p (IF3N), or C-terminal extension (IF3C), or both extensions (IF3NC). To ensure mitochondrial localization of hybrid proteins in the yeast cells, all of them were supplied with the MTS of Aim23p on their N-termini. The fourth protein constructed as the negative control was IF3 without MTS (IF3∆MTS). We again transformed the ∆AIM23 strain with plasmids coding for these proteins and revealed that clones containing IF3C are unstable while IF3N-containing yeast do not grow in glycerol medium at all. We decided to exclude these proteins from our analysis. The growth of the other strains is shown in Figure 3B.

As in our previous work [6], IF3 with MTS of Aim23p, when introduced in ∆AIM23 strain, slightly increases the rate of yeast growth in comparison with the parental strain. At the same time, the absence of MTS in IF3 leads to the growth rate equal to that of ∆AIM23 strain which we explain by the impossibility of the protein to be imported into mitochondria. When both Aim23p extensions are fused to IF3, the corresponding strain grows on glycerol medium with the rate equal to that of the wild-type strain. This means that adding Aim23p terminal extensions to *E. coli* IF3 makes the chimeric protein fully functional in yeast mitochondria. This finding indicates that the terminal extensions of Aim23p may have evolved as the “adaptors” that accommodate the translation factor of bacterial origin to the evolutionary changed protein biosynthesis system in mitochondria.

We also analyzed the rates of protein biosynthesis in mitochondria of mutant strains containing *E. coli* IF3 and its derivatives (Figure 3C). Calculations presented in Figure 3D show that, considering the biosynthesis rates of mitochondrial proteins most influenced by Aim23p (namely Cox1p, Cox2, and Atp9p), IF3NC ensures the rates similar to that of wild-type strain whereas IF3 in this regard is closer to ∆AIM23. Thus, Aim23p terminal extensions allow functioning of bacterial IF3 in translation in a way close to that of wild-type Aim23p in yeast mitochondria. 

### 2.3. Human mtIF3 Does Not Need Any Terminal Extension to Function Properly in Yeast Mitochondria

**Figure 4:**
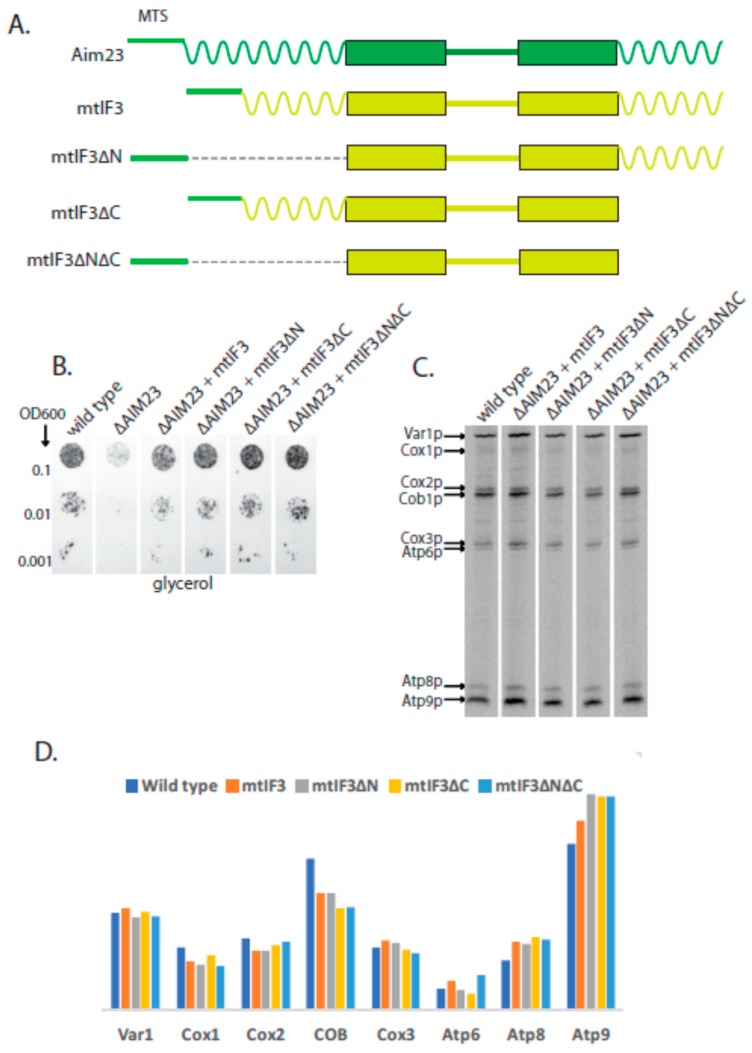
Human mtIF3 does not require any terminal extension to be active in yeast mitochondria. (**A**) Scheme of the hybrid proteins (the names are indicated on the left). The designations of the main structural parts and their colors are the same as in Figure 1. (**B**) The plate with glycerol-containing medium after 2 days of growth of the yeast strains indicated on the top. The ten-fold dilutions of the yeast suspensions were plated, starting from OD600 0.1 (indicated on the left). Wild-type: see Figure 2 legend. All other strains: D273-10B with the *AIM23* genomic disruption supplemented with the corresponding human mtIF3 version encoded by the plasmid. The experiment has been biologically repeated three times; the characteristic result is presented. (**C**) Separation of radioactively-labeled products of mitochondrial translation of the yeast strains indicated on the top. Cytosolic translation was inhibited by cycloheximide which was followed by adding of ^35^S-methionine incorporated exclusively in the mitochondrially-encoded proteins (for details, see Materials and Methods). The bands corresponding to all the eight individual proteins encoded in yeast mitochondria are visible; the proteins’ names are indicated on the left. (**D**) Estimated levels of the mitochondrially-encoded proteins (indicated on the bottom) after 15 min of labeling with ^35^S-methionine in the yeast strains indicated on the top.

Our next idea was to assess the necessity of human mtIF3 terminal extensions for its work in yeast mitochondria. We have shown earlier that the full-size human mtIF3 retains functionality of yeast mitochondria when synthesized in *S.cerevisiae* cells instead of Aim23p [6]. In this work, we constructed four chimeric proteins: human mtIF3 with its MTS being exchanged by MTS of Aim23p, (mtIF3 on Figure 4A), the same protein without N-terminal extension (mtIF3∆N), without C-terminal extension (mtIF3∆C), and without both extensions (mtIF3∆N∆C). The corresponding yeast strains were constructed on the basis of ∆AIM23 strain. These strains were tested for their growth on glycerol-containing medium (Figure 4B).

As in our previous studies, mtIF3 strain grew with the same rate as wild-type strain. Surprisingly, this was also true for all other mutant strains. Moreover, we were not able to detect any significant difference in individual protein synthesis rates in comparison with the wild type when analyzing ^35^S-methionine incorporation into mitochondrial translation products in discussed strains (Figure 4C,D), with the exception of cytochrome b which was slightly decreased in all strains, and may be Atp9 which was very slightly increased.This means that mammalian mtIF3 versions without one terminal extension or even without both of them are as functional in yeast mitochondria as wild-type Aim23p. This suggests that mammalian mtIF3 and Aim23p have quite diverged and their terminal extensions have different functions in mitochondriawhich is in a good agreement with our previous data [13].

## 3. Discussion

Translation initiation factors 3 from different organisms share the obvious common traits in their organization. The principal structure consists of two domains joined together by a flexible linker as it is in prokaryotes. We know that in vitro the isolated C-domain performs all activities of intact IF3, although having reduced affinity for 30S subunits. At the same time the isolated N-domain displays neither affinity for ribosomes nor a detectable function and it is supposed to modulate the interaction of the IF3 with the ribosome and to be involved in IF3 recycling [21]. IF3s in the mitochondria retain the basic structural blocks including the two domains and a linker, additionally complemented with the mitochondria-specific N- and C-terminal extensions. This work aims to study the mitochondria-specific regions of the S.cerevisiae mitochondrial translation initiation factor 3, Aim23p.

To investigate this subject, we exploited the setup based on in vivo experiments with the IF3s from the different organisms, namely Aim23p (yeast mitochondrial IF3, the main object), *E. coli* IF3 (prokaryotic ancestor without the terminal extensions) and human mitochondrial IF3 (mitochondrial homologue with the terminal extensions). We engineered the yeast strains expressing the chimeric proteins in which the N- and C-terminal extensions were either deleted or switched between each other and compared their growth rates and mitochondrial translation products.

We demonstrated that Aim23p requires only one terminal extension to be fully functional in yeast mitochondria. This finding somewhat reminds the situation when only one domain of bacterial IF3 was sufficient to fulfill its activity. The only significant difference is that there is no matter which exact extension of Aim23p is absent if another one remains untouched. However, the deletion of both extensions produces the *AIM23* gene deletion phenotype meaning that the protein is no longer functional in yeast cells. Previously we showed that bacterial IF3 could complement *AIM23* deletion, but only partially with the growth rate being considerably slower when of the wild-type strain [6]. On the other hand, deletions of the extensions do not alter the catalytic core domain of the Aim23p. Taken together, these results make us propose the model according to which the terminal extensions serve to fit the catalytic core of initiation factor 3 to the mitochondrial ribosome. Bacterial IF3 poorly fits the mitochondrial ribosome explaining why the complementation of the deletion is only partial. In the same way, Aim23p devoid of its N- and C-terminal extensions is unable to find its proper place on the ribosome and fulfill its role despite the preservation of the catalytic domains in both proteins.

We went further to show that the fusion of N- and C-terminal extensions of the yeast Aim23p to the bacterial IF3 makes the protein fully functional in yeast mitochondria. This chimeric IF3 displays significantly higher activity than the normal bacterial protein. This observation supports our model, suggesting that the extensions serve to fit the catalytic core of the IF3 on the mitochondrial ribosome correctly.

Finally, we exploited the same strategy to investigate if the extensions of Aim23p could make the human mitochondrial IF3 functional in yeast cells. However, all versions of human mtIF3 that we tested were functional in yeast mitochondria. The most surprising finding was that human mtIF3 is functional in yeast mitochondria even without both extensions. It demonstrates that human factor may function differently from its bacterial and yeast homologues and its extensions may play different roles than those of Aim23p. Some differences between Aim23p and human mtIF3 with regard to their extensions have been hypothesized in in silico analyses. Aim23p terminal extensions are substantially longer than those of human mtIF3 [13], and molecular modeling points that N-terminal extension of Aim23p (the longest one) might be of helical structure [22] while human mtIF3 extensions are believed to be unstructured [18].

Basically, the lack of necessity of human mtIF extensions for protein function in yeast neither supports our model nor produces the arguments against it. The discussed phenomenon can be attributed to the general differences in mitochondrial translation initiation in yeast and mammals.

## 4. Materials and Methods

### 4.1. Plasmids, Strains, Oligonucleotides

The full lists of plasmids, strains, and oligonucleotides used in this work can be found in Table 1, Table 2 and Table 3, respectively.

### 4.2. Cloning, Yeast Strains Construction, and Standard Procedures

Different versions of *AIM23* (*S. cerevisiae*), *MTIF3* (*H. sapiens*) and *infC* (*E. coli*) genes, as well as the fusion genes containing different parts of above-mentioned genes, were cloned into a pRS317 vector by a standard PCR-restriction-ligation approach using overlap extension PCR [23]. To ensure proper expression in yeast, all pRS317 inserts contained 5’- and 3’-flanking regions of *AIM23* gene. All resulting plasmids were verified by Sanger sequencing.

Genomic disruption of *AIM23* gene was performed using KanMX4 cassette containing geneticin (G418) resistance gene. This cassette was obtained by PCR with oligonucleotides that contained 5′- and 3′-proximal parts complementary to the upstream and downstream regions of *AIM23* gene, respectively, for homologous recombination. Yeast haploid strain D273-10B was transformed by KanMX4 cassette as per [24]. Transformants were selected on the medium with G418 (Thermo Fischer Scientific, Waltham, MA, USA). Screening of the G418-resistant clones was performed by PCR. The resulting ∆AIM23 strain was checked for the characteristic phenotype detected earlier [15] (delay of growth on glycerol-containing medium). To produce the strains where *AIM23* gene was replaced by one of its mutant versions, the ∆AIM23 strain was transformed by the corresponding plasmids mentioned above, again as per [24]. Transformants were selected on the medium without lysine.

Western-blot was performed by standard protocol using the rabbit antibodies against 6-His-tagged recombinant Aim23p (produced on our order by Almabion, York, UK) and commercial antibodies against Por1p (Abcam, Cambridge, UK).

### 4.3. Measurement of the Mitochondrial Translation

Labeling of yeast mitochondrially-synthesized proteins with ^35^S-methionine was carried out in whole cells that were cultured in medium containing 2% of galactose as carbon source up to 2–3 units of OD_600_. The cells were incubated for 5 min at 30 °C in the presence of 0.2 mg/mL cycloheximide in order to inhibit cytosolic translation. Immediately after this, 25–30 µCi of ^35^S-methionine (Perkin Elmer, Waltham, MA, USA) was added, and incubation continued for 20 more minutes at 30 °C. After incorporation of the label into the products of mitochondrial translation, equal amounts of total cell proteins were separated on a 17.5% PAGE, subjected to autoradiographic analysis (using Storm scanner, GE Healthcare, Chicago, IL, USA). The results were quantified using ImageJ software (Version 1.50B, National Institutes of Health, Bethesda, Maryland, USA) [25].

## Figures and Tables

**Table 1:** Plasmids used.

Plasmid	Description
pAim23	pRS317 with cloned *AIM23* gene
pAim23∆MTS	pRS317 with cloned *AIM23* gene lacking mitochondrial targeting sequence
pAim23∆N	pRS317 with cloned *AIM23* gene lacking N-terminal extension
pAim23∆C	pRS317 with cloned *AIM23* gene lacking C-terminal extension
pAim23∆N∆C	pRS317 with cloned *AIM23* gene lacking N- and C-terminal extensions
pIF3	pRS317 with cloned *infC* gene from *E. coli* (IF3-coding) fused with the Aim23p mitochondrial targeting sequence
pIF3∆MTS	pRS317 with cloned *infC* gene
pIF3N	pRS317 with cloned *infC* gene fused with the Aim23p N-terminal extension
pIF3C	pRS317 with cloned *infC* gene fused with the Aim23p C-terminal extension
pIF3NC	pRS317 with cloned *infC* gene fused with the Aim23p N- and C-terminal extensions
pmtIF3	pRS317 with cloned *MTIF3* gene from *Homo sapiens* (mtIF3-coding) fused with the Aim23p mitochondrial targeting sequence
pmtIF3∆N	pRS317 with cloned *MTIF3* gene lacking N-terminal extension
pmtIF3∆C	pRS317 with cloned *MTIF3* gene lacking C-terminal extension
pmtIF3∆N∆C	pRS317 with cloned *MTIF3* gene lacking N- and C-terminal extensions

All generated in this work on the base of pRS317, yeast shuttle vector with *LYS2* marker gene.

**Table 2:** *S. cerevisiae* strains used in the work.

Strain	Genotype/Description
∆AIM23	MATa mal (lys2, ura3) AIM23::KanMX4 D273-10B DUL2 with *AIM23* genomic disruption
Aim23 (“wild type” on Figures)	∆AIM23 + pAim23
Aim23∆MTS	∆AIM23 + pAim23∆MTS
Aim23∆N	∆AIM23 + pAim23∆N
Aim23∆C	∆AIM23 + pAim23∆C
Aim23∆N∆C	∆AIM23 + pAim23∆N∆C
IF3	∆AIM23 + pIF3
IF3∆MTS	∆AIM23 + pIF3∆MTS
IF3N	∆AIM23 + pIF3N
IF3C	∆AIM23 + pIF3C
IF3NC	∆AIM23 + pIF3NC
mtIF3	∆AIM23 + pmtIF3
mtIF3∆N	∆AIM23 + pmtIF3∆N
mtIF3∆C	∆AIM23 + pmtIF3∆C
mtIF3∆N∆C	∆AIM23 + pmtIF3∆N∆C

All generated in this work on the basis of D273-10B DUL2 strain (MATa mal (lys2, ura3), kindly gifted by Thomas Fox, Cornell University, USA), except for ∆AIM23 which was produced earlier [15].

**Table 3:** Oligonucleotides used in the work.

1	*AIM23* gene cloning into pRS317	gcatAAGCTTggctatcatgcatccattg
2		gcaTCTAGAcagcatttcggggcaac
3	AIM23∆MTS cloning into pRS317: producing first (5’) PCR-product for further OE-PCR	gcatAAGCTTggctatcatgcatccattg
4		atccacggacctgatgtt
5	AIM23∆MTS cloning into pRS317: producing second (3’) PCR-product for further OE-PCR	aggtccgtggatATGaatgcatcatctaccacag
6		gcaTCTAGAcagcatttcggggcaac
7	AIM23∆N cloning into pRS317: producing first (5’) PCR-product for further OE-PCR	gcatAAGCTTggctatcatgcatccattg
8		gtctctgctgaagtattttg
9	AIM23∆N cloning into pRS317: producing second (3’) PCR-product for further OE-PCR	tacttcagcagagactggagcaccgggacag
10		gcaTCTAGAcagcatttcggggcaac
11	AIM23∆C cloning into pRS317: producing first (5’) PCR-product for further OE-PCR	gcatAAGCTTggctatcatgcatccattg
12		tggtttaacgtcctttggta
13	AIM23∆C cloning into pRS317: producing second (3’) PCR-product for further OE-PCR	aaaggacgttaaaccataaatagaagcaaatgacatcag
14		gcaTCTAGAcagcatttcggggcaac
15	AIM23∆N∆C cloning into pRS317: producing third (central) PCR-product for further OE-PCR	tacttcagcagagactggagcaccgggacag
16		tggtttaacgtcctttggta
17	IF3 versions cloning into pRS317: producing IF3 for further OE-PCRs	aaaggcggaaaacgagttc
18		ctgtttcttcttaggagcg
19	IF3 versions cloning into pRS317: fusing *AIM23* 5’-flank with IF3 for further OE-PCR	gcatAAGCTTggctatcatgcatccattg
20		cgttttccgcctttcatatccacggacctgatg
21	IF3 versions cloning into pRS317: fusing *AIM23* 5’-flank and MTS with IF3 for further OE-PCR	gcatAAGCTTggctatcatgcatccattg
22		cgttttccgcctttgtctctgctgaagtattttgt
23	IF3 versions cloning into pRS317: fusing *AIM23* 5’-flank, MTS, and N-terminal extension with IF3 for further OE-PCR	gcatAAGCTTggctatcatgcatccattg
24		cgttttccgcctttagtaatcaggatttttttcctg
25	IF3 versions cloning into pRS317: fusing *AIM23* 3’-flank and C-terminal extension with IF3 for further OE-PCR	tcctaagaagaaacagcaaaacaacgataagagggc
26		gcaTCTAGAcagcatttcggggcaac
27	IF3 versions cloning into pRS317: fusing *AIM23* 3’-flank with IF3 for further OE-PCR	Tcctaagaagaaacagtaaatagaagcaaatgacatcagaat
28		gcaTCTAGAcagcatttcggggcaac
29	mtIF3 versions cloning into pRS317: fusing *AIM23* 5’-flank and MTS with mtIF3	gcatGGGCCCggctatcatgcatccattg
30		tgtgctggtgctgtgtctctgctgaagtattttg
31	mtIF3 versions cloning into pRS317: producing mtIF3 without MTS for further OE-PCR	acagcaccagcacagttg
32		gtcatttgcttctatttactgatgcagaacatttgattc
33	mtIF3 versions cloning into pRS317: fusing *AIM23* 3’-flank with mtIF3	taaatagaagcaaatgacatca
34		gcaTCTAGAcagcatttcggggcaac
35	mtIF3∆N cloning into pRS317: producing first (5’) PCR-product for further OE-PCR	gcatGGGCCCggctatcatgcatccattg
36		ccttcattctgggtgtctctgctgaagtattttg
37	mtIF3∆N cloning into pRS317: producing second (3’) PCR-product for further OE-PCR	acccagaatgaaggaaaaaag
38		gcaTCTAGAcagcatttcggggcaac
39	mtIF3∆C cloning into pRS317: producing first (5’) PCR-product for further OE-PCR	gcatGGGCCCggctatcatgcatccattg
40		tttgctgaaagcacgaagaa
41	mtIF3∆C cloning into pRS317: producing second (3’) PCR-product for further OE-PCR	cgtgctttcagcaaataaatagaagcaaatgacatcag
42		gcaTCTAGAcagcatttcggggcaac
43	mtIF3∆N∆C cloning into pRS317: producing third (central) PCR-product for further OE-PCR	acccagaatgaaggaaaaaag
44		tttgctgaaagcacgaagaa
45	Screening of pRS317-based constructs	gtaaaacgacggccagt
46		ggaaacagctatgaccatg

All synthesized by Evrogen. Restriction sites are in capital letters.
